# The influence of twist angle on the electronic and phononic band of 2D twisted bilayer SiC

**DOI:** 10.1039/d3ra04525k

**Published:** 2023-11-06

**Authors:** Hoa Van Nguyen, Phi Minh Nguyen, Vi Toan Lam, Sugino Osamu, Hanh Thi Thu Tran

**Affiliations:** a Laboratory of Computational Physics, Faculty of Applied Science, Ho Chi Minh City University of Technology (HCMUT) 268 Ly Thuong Kiet Street, District 10 Ho Chi Minh City Vietnam thuhanhsp@hcmut.edu.vn; b Vietnam National University Ho Chi Minh City Linh Trung Ward, Thu Duc District Ho Chi Minh City Vietnam; c The Institute for Solid State Physics, The University of Tokyo Kashiwa Chiba 277-8581 Japan

## Abstract

Silicon carbide has a planar two-dimensional structure; therefore it is a potential material for constructing twisted bilayer systems for applications. In this study, DFT calculations were performed on four models with different twist angles. We chose angles of 21.8°, 17.9°, 13.2°, and 5.1° to estimate the dependence of the electronic and phononic properties on the twist angle. The results show that the band gap of bilayer SiC can be changed proportionally by changing the twist angle. However, there are only small variations in the band gaps, with an increment of 0.24 eV by changing the twist angle from 5.1° to 21.8°. At four considered twist angles, the band gaps decrease significantly when fixing the structure of each layer and pressing the separation distance down to 3.5 Å, 3.0 Å, 2.7 Å, and 2.5 Å. A noteworthy point is that the pressing also makes the band linearly smaller at a certain rate regardless of the twist angles. Meanwhile, the phonon bands are not affected by the value of the twist angle. The optical bands are between 900 cm^−1^ and 1100 cm^−1^ and the acoustic bands are between 0 cm^−1^ and 650 cm^−1^ at four twist angles.

## Abstract 2

The height of atoms in the upper and lower layers of 5.1° (a and b), 13.2° (c and d) and 21.8° (e and f) twisted models. The interlayer distances of peaks tend to decrease as the twist angle increases.

## Graphical abstract

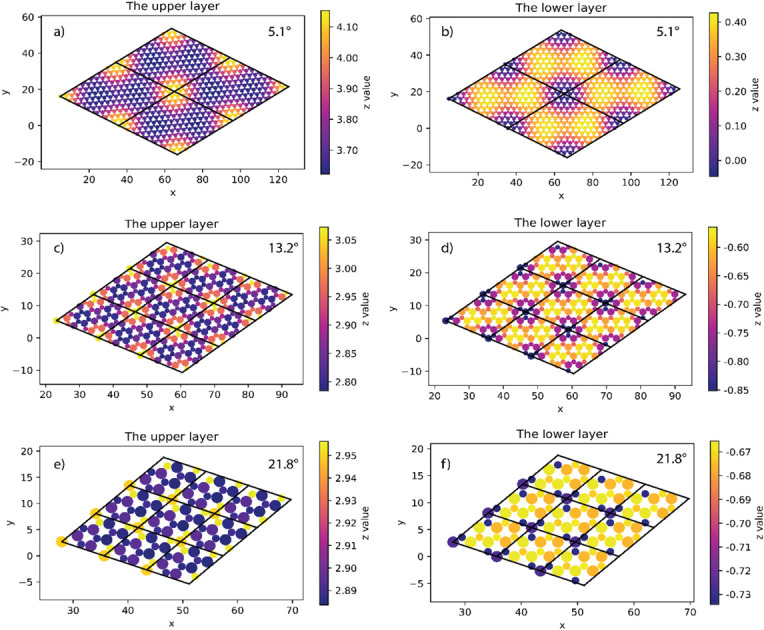

## Introduction

Thanks to the successful preparation of graphene, two-dimensional (2D) honeycomb structures have attracted a great deal of research interest in recent years.^1,2^ Over the past two decades, many research groups have investigated various honeycomb-structured materials, such as single-walled and multi-walled carbon nanotubes and fullerene spheres.^3^ In the last decade, twisted bilayer graphene (TBLG) has become an interesting topic for many researchers due to its novel phenomena that a single graphene layer does not have.^4–10^ One of the most extraordinary discoveries about the TBLG is the “magic angle” of 1.1°, where the TBLG becomes a superconductor with nearly flat bands.^4,7,11,12^

Based on this, many studies have attempted to investigate the twisted bilayer structure of other twisted 2D materials, such as black phosphorene,^13^ blue phosphorene,^14^ and molybdenum disulfide.^15^ This research has shown that twisted bilayer structures can alter the band structure, with different twist angles affecting the bandwidth and causing variations in the direct and indirect band gaps. Changing the band gap results in changing the sensitivity of the material to photons. Thus, the twisted bilayer systems are commonly used in optoelectrical devices such as photosensors/photodetectors,^16–19^ transistors^20,21^ and even solar cells.^22,23^

In addition to the above-mentioned buckled 2D materials, silicon carbide (SiC) has attracted the attention of many research groups due to its outstanding properties.^24–26^ The 2D SiC is a semiconductor with a wide band gap of 2.30–2.55 eV, but in the nanoribbon form, SiC behaves as a zigzag-type metal and an armchair-type semiconductor.^27,28^ Different from the buckled 2D materials, silicon carbide (SiC) has a planar structure with sp^2^-characterized bonding.^29^ SiC is also used as a base to integrate untwisted and twisted bilayer heterostructures, which include two layers of two different 2D materials.^30–32^ The graphene-like planar structure and the band gap flexibility response to stress or strain^33^ make SiC an ideal conventional material for the multilayer twist-based applications.

In general, these research results show that the stacking and twisting bilayer is a promising area to improve the performance of electronic, photonic and phononic properties of SiC. This motivates our team to investigate the twisted bilayer SiC (TBSC). We used the density functional theory (DFT) approach to study the fundamental properties of TBSC *via* the twist angle. We calculated the electronic, phonon band with respect to four twist angles: 21.8°, 17.9°, 13.2° and 5.1°, and extracted the change tendency of band gap and phonon band when the twist angle or interlayer distance changes. The results clearly show that the twisting and pressing bilayer can tune the electronic character of TBSC, but not the phonon band.

## Calculation method

### General method

**Fig. 1:**
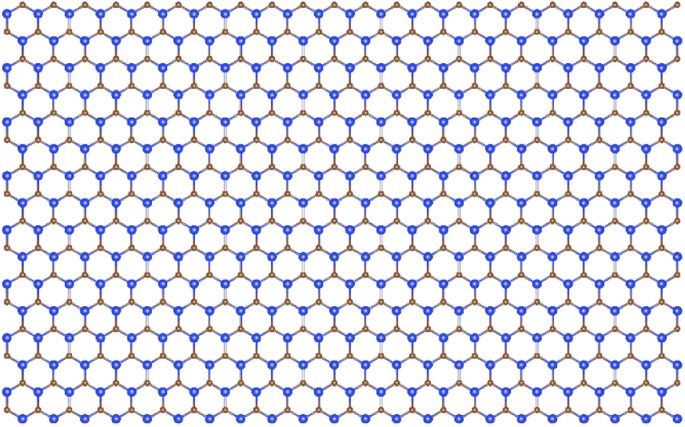
The SiC monolayer with 704 atoms, (Si atoms are blue, C atoms are grown).

**Fig. 2:**
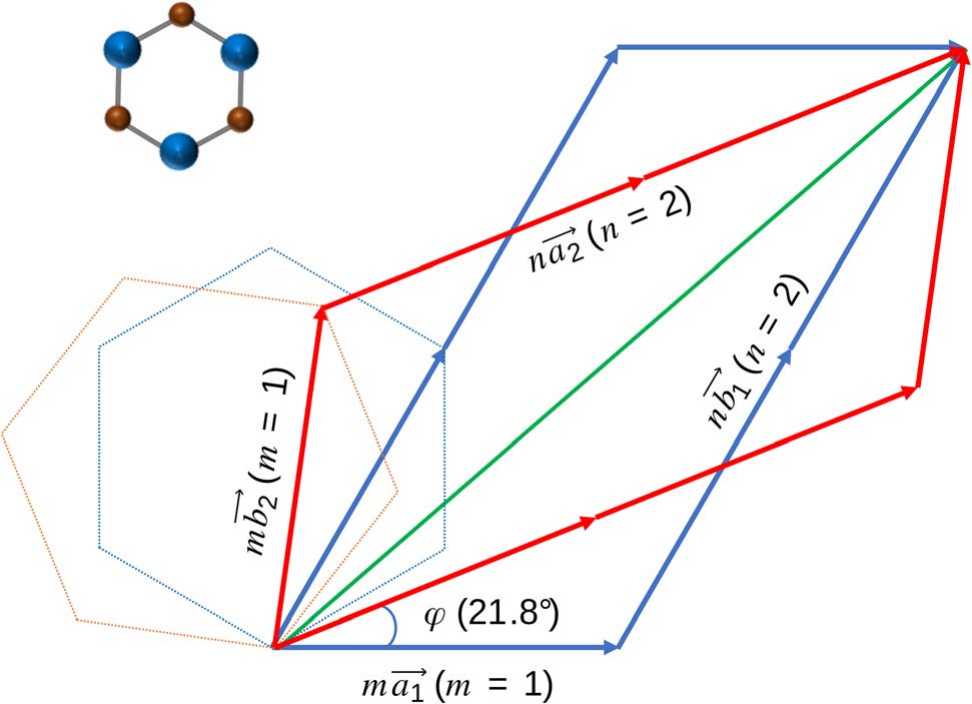
The closest repeated point in the bilayer model with the twist angle of 21.8°.

We used first-principles calculations to study the effect of twist angle on the electronic and phonon properties of bilayer silicon carbide. We used the Spanish Initiative for Electronic Simulations with Thousands of Atoms (SIESTA) software^34,35^ to perform our DFT calculation. First, we constructed a 704-atom SiC monolayer with the Si–C bond length of 1.79 Å^36,37^ and obtained the relaxed coordinates of the lattice (Fig. 1). Then, we stacked two relaxed SiC layers with respect to the AA-stacked configuration and rotated them by a certain relative angle. In all cases, we chose the Si–Si stacking position as the origin of twisting (see Fig. 2). We found some special twist angles that allow us to extract the respective unit cells of these systems with an appropriate number of atoms (see the following section for details). At each twist angle we analyzed the energy and phonon band structures of the relaxed and fixed unit cell.

To estimate the influence of the interlayer distance on the electronic band structure, we performed the DFT calculations on the SiC bilayer systems with different interlayer distances. The initial separation distance of the two SiC layers is 2.22 Å. After relaxation and obtaining the optimized separation distance, we set up several unrelaxed unit cells with all atoms fixed and decrease the separation distances until the twisted SiC bilayer complex becomes a conductor.

### DFT parameters

SIESTA is based on the linear combination of atomic orbitals.^34,35^ The exchange–correlation functional used in the DFT calculation is the generalized gradient approximation of Perdew, Burke, and Ernzerhof.^38^ It is well known that the GGA-PBE functional generally underestimates the dispersive interactions, so the calculated band gap is also underestimated.^39–41^ Thereby, higher-level theories, hybrid functionals, or GW method are usually conducted to obtain the more precise band gap value.^41^ However, the band structure is marginally affected by the dispersive interactions and this study mainly focuses on the variation of the band gap. Thus, the GGA-PBE exchange–correlation function is still suitable for adjusting the different band gaps between models.

The pseudopotential used is generated by the improved Troullier–Martins scheme^42^ with three core and four valence orbitals. We also implied the double zeta polarized with the split norm of 0.3. The *k*-point grid is generated from the Monkhorst–Pack (MP) scheme. In the SiC monolayer relaxation, we used the *k*-point grid of (3 × 3 × 1), but in all twisted bilayer models the (5 × 5 × 1) grid was used. The grid cutoff energy is 200 Ry and the electronic temperature in the Dirac–Fermi function is 300 K. To obtain the optimized structure, we set the force tolerance to 0.02 eV Å^−1^ for the conjugate gradient run. To obtain the phonon band, we used the relaxed coordinations of each twisted model and set the atomic displacement of 0.04 bohr radius. From the force-constant matrix, we deduced the phonon frequencies by using the package Vibra of SIESTA.

### Twist angle

Obviously, the new lattice vectors must satisfy the inclusive lattice vectors of both layers and are chosen to be as small as possible. In other words, the new lattice vectors are the least common multiple of the inclusive lattice vectors. So, to find the unit cell of the twisted bilayer model, we started by calling the lattice vectors of the first layer as 
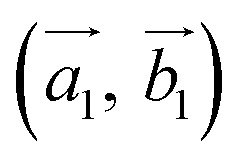
 (the blue arrows in Fig. 2) and the upper layer as 
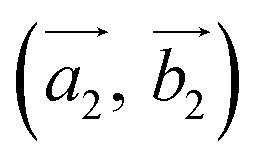
 (the red arrows in Fig. 2). We determined the unit cell of both layers by finding the closest repeating points of the origin. Such a point would satisfy the equation1
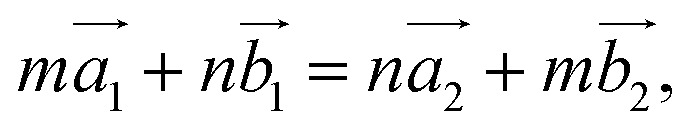
where *m* and *n* are the smallest possible integers. Transforming eqn (1), we get the ratio2
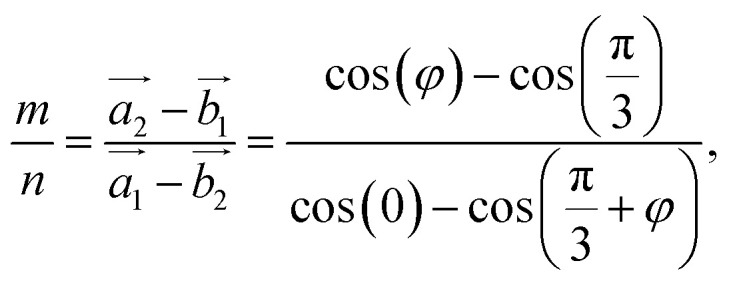
with *φ* as the twist angle.

For each value of *φ*, we calculated the value of the right-hand side in eqn (2). Then we tried different values of *m* and *n*, but not more than 8 to limit the size of the unit cells. If the error between the right-hand side in eqn (2) and the *m*/*n* value is less than 0.001, we will record this twist angle *φ*. By gradually increasing the twist angle, we repeated these steps and found certain values of *φ*, *m*, and *n* (listed in Table 1). These values are suitable not only for SiC bilayer, but also for all materials with hexagonal 2D structure. Because of the symmetry of the SiC layer, we only considered the *φ* values in the 0°–30° range. In this study, we investigated four twist angles, including 5.1°, 13.2°, 17.9°, and 21.8°, which refer to the largest and three smallest unit cells shown in Table 1.

**Table 1:** The appropriate values of *φ* and the respective *m*,*n*

*φ* (°)	*m*	*n*	Number of atom
5.1	6	7	508
6.0	5	6	364
9.4	3	4	148
11.0	5	7	436
13.2	2	3	76
16.4	3	5	196
17.9	4	7	124
21.8	1	2	28
26.0	3	7	316
27.8	2	5	52

## Results and discussion

### The warp of relaxed twisted bilayer structure

**Fig. 3:**
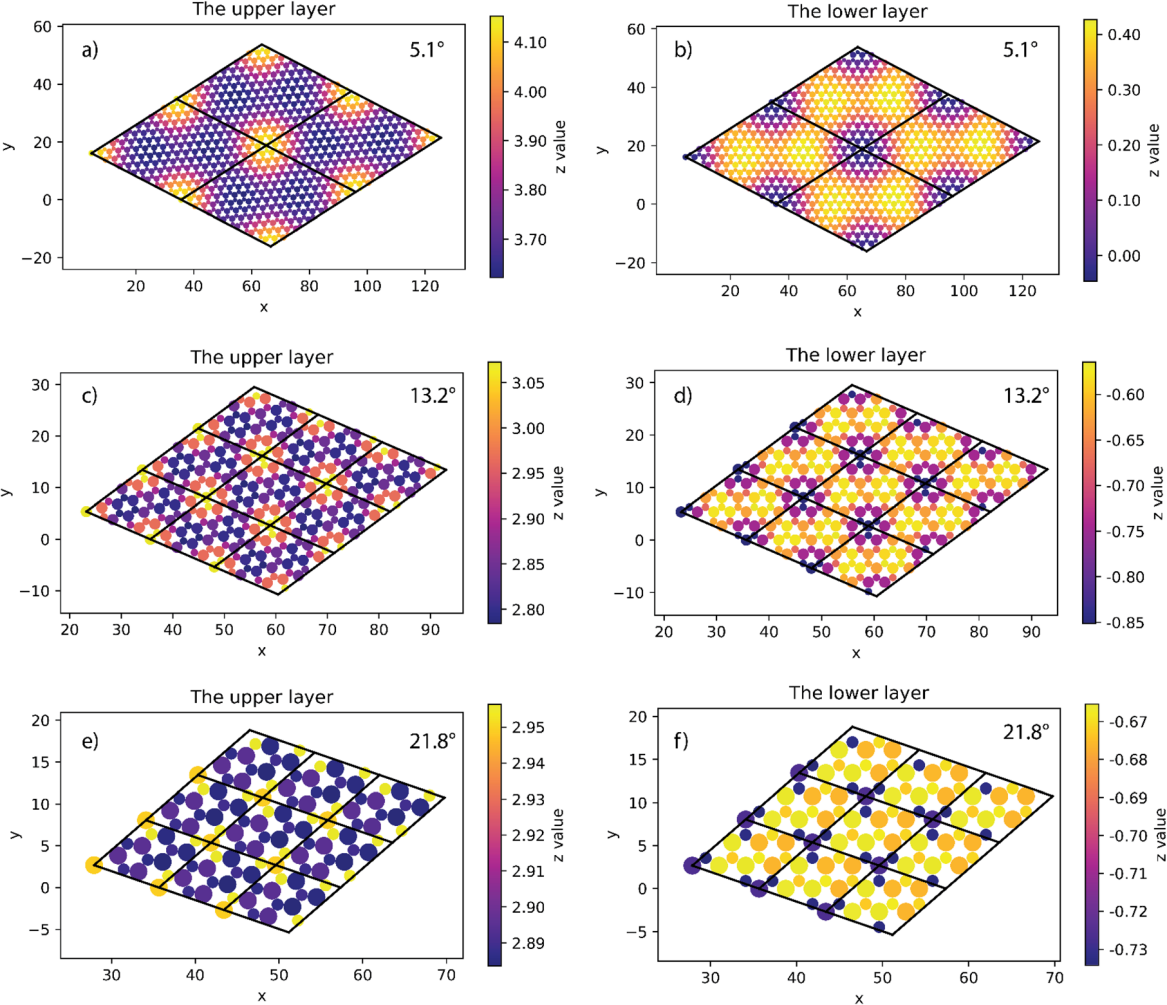
The height of atoms in the upper and lower layers of 5.1° (a and b), 13.2° (c and d) and 21.8° (e and f) twisted model.

**Fig. 4:**
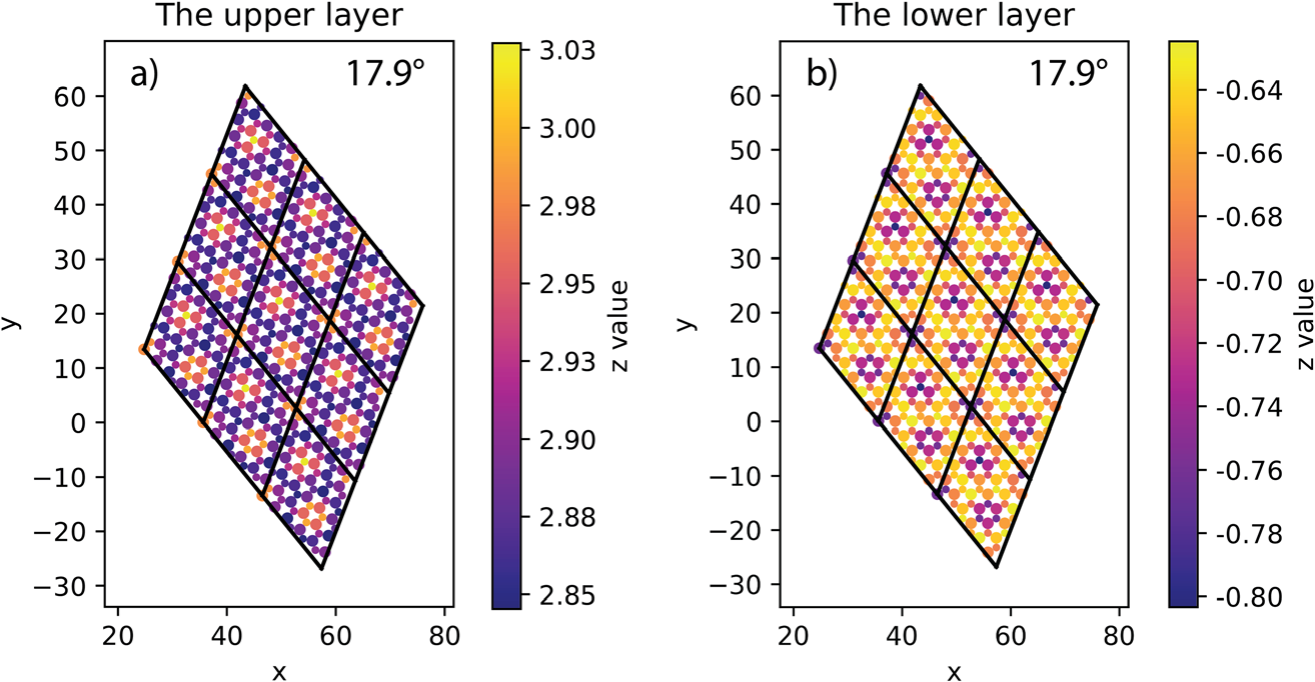
The height of atoms in the upper (a) and lower layer (b) of 17.9° twisted model.

At all angles considered, the relaxed structures of the twisted SiC bilayer are warped like the twisted 2D bilayer graphene^43^ (see Fig. 3 and 4). The warps obtained with respect to the twist angles of 21.8°, 13.2° and 5.1° are similar to each other. The top and bottom SiC layers have the symmetrical structure. At each corner of these unit cells, the upper layer has an upward peak, and the lower layer has a downward peak. In the upper layers, the atoms are gradually shifted downward as they move away from the peaks, creating two symmetrical concavities as shown in Fig. 3. According to the color shown in Fig. 3, the warp intensity is found to be similar in all the three models.

The situation is, however, different for the 17.9° twist angle model. In addition to the corners, there are two more peaks that emerge inside the unit cell (as shown in Fig. 4). It is worth noting that all peaks are paired and symmetrical. The peaks are always outward facing and are only found where the same sites in the SiC hexagon of both layers are stacked. Because we have chosen the Si atom as the origin of the rotation, the Si atoms in four corners are directly stacked, forming four peaks each. In this model (*i.e.*, the 17.9° twist angle model) the two peaks inside the unit cell are related to the stack of carbon atoms (at *x* = 32.5 and *y* = 19.7 in Fig. 4) and the centers of the hexagons (at *x* = 34.1 and *y* = 9.9 in Fig. 4). In addition, the optimized bilayer separation distance increases as the twist angle decreases (see Table 2, Fig. 6). This tendency is consistent with the case of twisted bilayer blue phosphorene,^22^ where the distance between two blue phosphorene layers decreases with the relative twist angle. Although that research^22^ chose the AB-stacked configuration for twisting, the repeated AA stacking was also found in the moiré pattern. Thus, there is an equivalence between AA and AB stacking in the twisted bilayer systems. In addition, when the twist angle changes from 5.1° to the untwisted state, the interlayer distance slightly decreases by 0.11 Å. This could be the result of the disappearance of the moiré pattern and the layers are no longer warped.

**Table 2:** The optimized separate distances of bilayer with respect to the twist angles. These values are represented by the Si–Si distance at the origin of rotation

Twist angle (°)	Optimized separation distance (Å)
21.8	3.67
17.9	3.74
13.2	3.92
5.1	4.19
0.0	4.084

### The band structure of relaxed twisted bilayer SiC

**Fig. 5:**
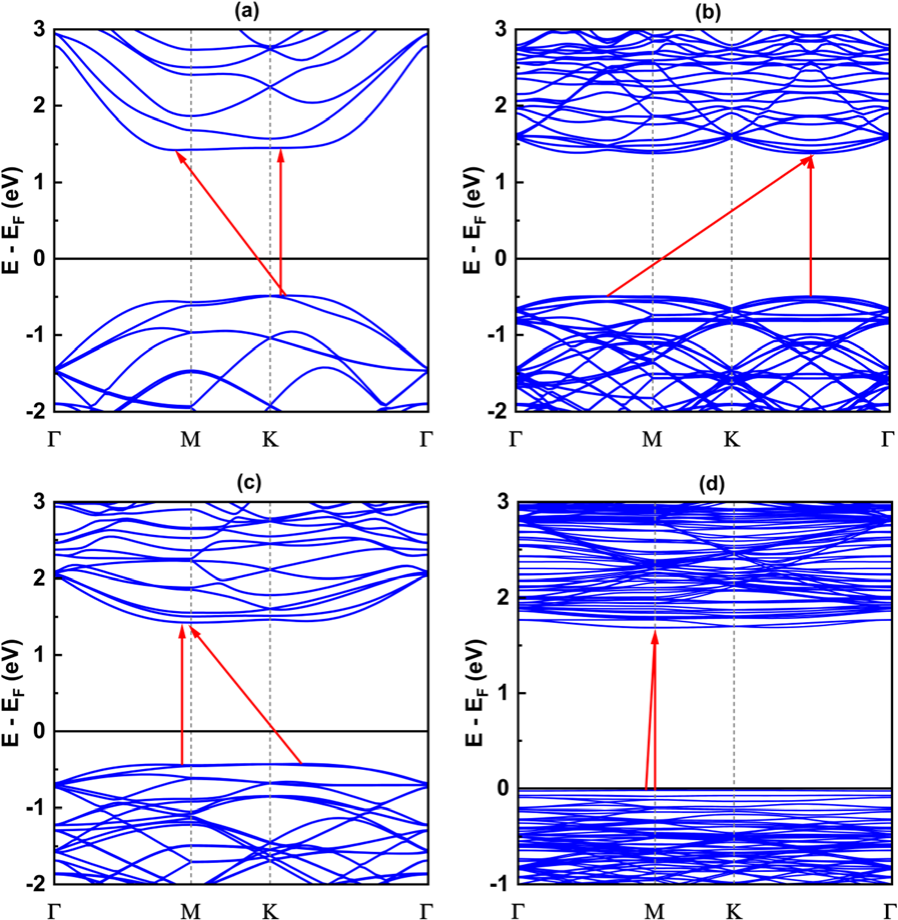
The band structures of relaxed configurations with respect to four twist angles: (a) 21.8°, (b) 17.9°, (c) 13.2° and (d) 5.1°. The Fermi energies are used for the offset level, which are −4.46 eV, −4.45 eV, −4.5 eV and −4.79 eV for 21.8°, 17.9°, 13.2 and 5.1° case, respectively.

**Fig. 6:**
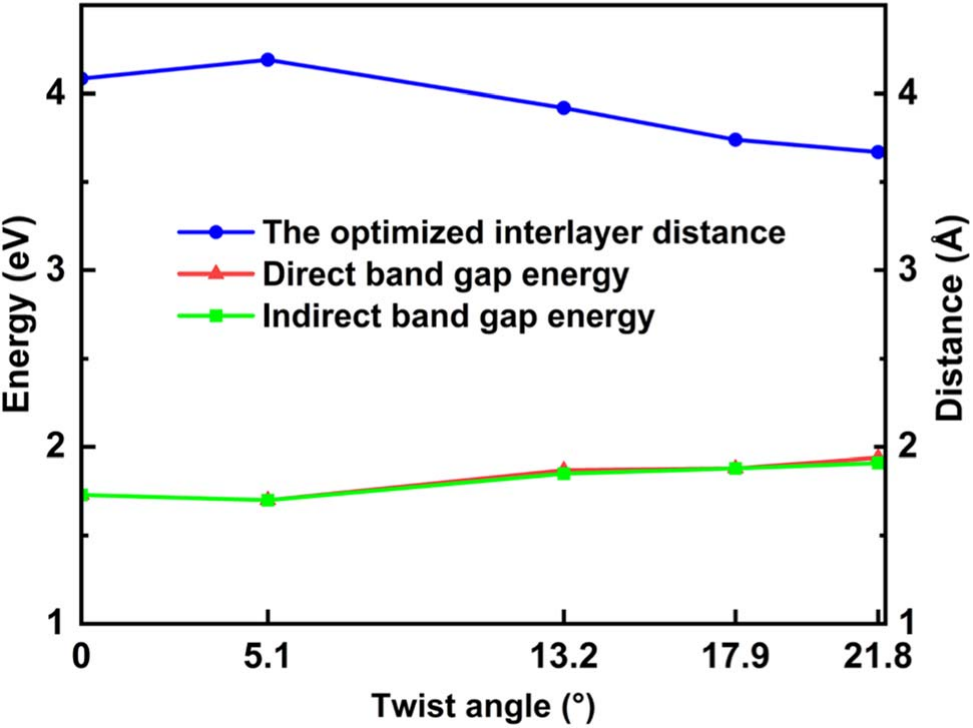
The values of direct and indirect band gaps and the optimized interlayer distance with respect to the twist angles.

The electronic band structures shown in Fig. 5, 6 and Table 3 illustrate the variation of energy levels with twist angle. As a result of increasing the number of atoms in the unit cell, the band is found to be densest in the 5.1° case and less dense in the 17.9°, 13.2°, and 21.8° cases, in that order. The bands just above and below the Fermi level are flat in all studied models, which has already been observed in other twisted materials.^22,44^ As a result, the direct and indirect band gaps are not so different from each other. The value is only slightly reduced as the twist angle decreases, as shown in Table 3 and Fig. 6. This indicates that twisting may not be an efficient method to tune the semiconducting property of the SiC bilayer.

**Table 3:** The direct and indirect band gap energy of the relaxed models with different twist angles

Twist angle (°)	Direct band gap energy (eV)	Indirect band gap energy (eV)
21.8	1.94	1.91
17.9	1.88	1.88
13.2	1.87	1.85
5.1	1.70	1.70
0.0	1.73	1.73

**Fig. 7:**
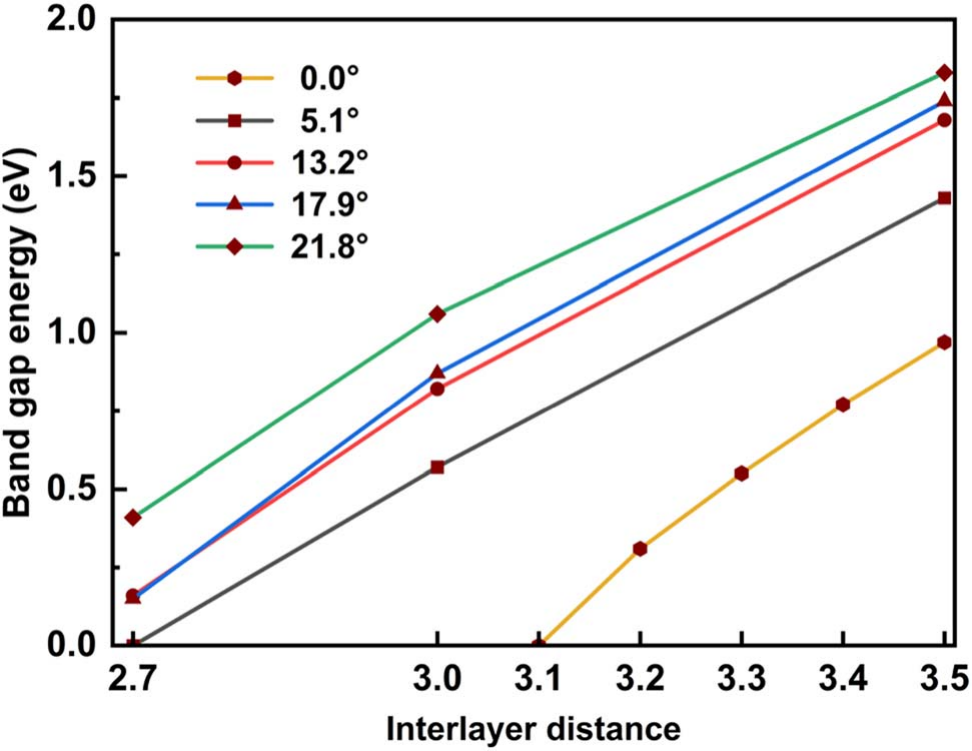
The relation between the band gap energy and the interlayer distance of twisted and untwisted SiC bilayer.

In addition, for a comprehensive commentary, we also considered the influence of the interlayer distance on the bandgap, which is shown in Table 4 and Fig. 7. When the interlayer distance is taken to be the same, the bandgap decreases as the twist angle decreases, just as in the case of optimized distance. In addition, compressing the bilayer models also causes the band gap to decrease. When the interlayer distance decreases to 2.7 Å, the bandgap becomes vanishes for the 5.1° case and is finite but less than 0.5 eV for other cases, meaning that the twisted SiC bilayer complexes become conductors. This variation is also observed in the case of zero twist angle (untwisted). Because the zero twist angle structure has smaller values of band gap, we calculated at different points of interlayer distance to show the tendency, and the result is approximated to a linear decrement (shown in Table 5 and Fig. 7).

**Table 4:** The dependence of direct band gap (eV) on the interlayer distance. The dashes represent the band gap energies which are smaller than 0.15 eV or don't exist, meaning that they are conductors

Twist angle (°)	Interlayer distance			
	3.5 (Å)	3.0 (Å)	2.7(Å)	2.5 (Å)
21.8	1.83	1.06	0.41	—
17.9	1.74	0.87	0.15	—
13.2	1.68	0.82	0.16	—
5.1	1.43	0.57	—	—

**Table 5:** The dependence of direct band gap (eV) on the interlayer distance for the zero twist angle (untwisted). The dashes represent the band gap energies which are smaller than 0.15 eV or don't exist, meaning that they are conductors

Twist angle (°)	Interlayer distance				
	3.5 (Å)	3.4 (Å)	3.3 (Å)	3.2 (Å)	3.1 (Å)
0.0	0.97	0.77	0.55	0.31	—

The increase in band gap is proportional to the increase in twist angle and interlayer distance. It may indicate that there is discernible charge transfer between the layers. Because the transfer of charge between the layers will induce electrostatic interactions between the layers, thereby changing the energy level. When the charge from the lower layer transfers to the upper layer, the electrostatic potential of the upper layer will increase, and the electrostatic potential of the lower layer will decrease. This results in the energy level of the conduction band (upper layer) moving up, and the energy level of the valence band moving down. Therefore, the band gap increases. As the twist angle and interlayer distance increase, the overlap between the layers decreases, and charge transfer increases, so the band gap increases further.

In general, this effect of the interlayer spacing squeeze on the band gap is more significant than the twist angle decrement. These results may suggest a theoretical way to tune the conductivity property of SiC bilayer by twisting and pressing. Furthermore, our results show an almost linear relationship between the band gap energy and the interlayer distance (shown in Fig. 7). A notable point is that the band gap decrement has a similar rate in all twist angle models.

### The phonon band

**Fig. 8:**
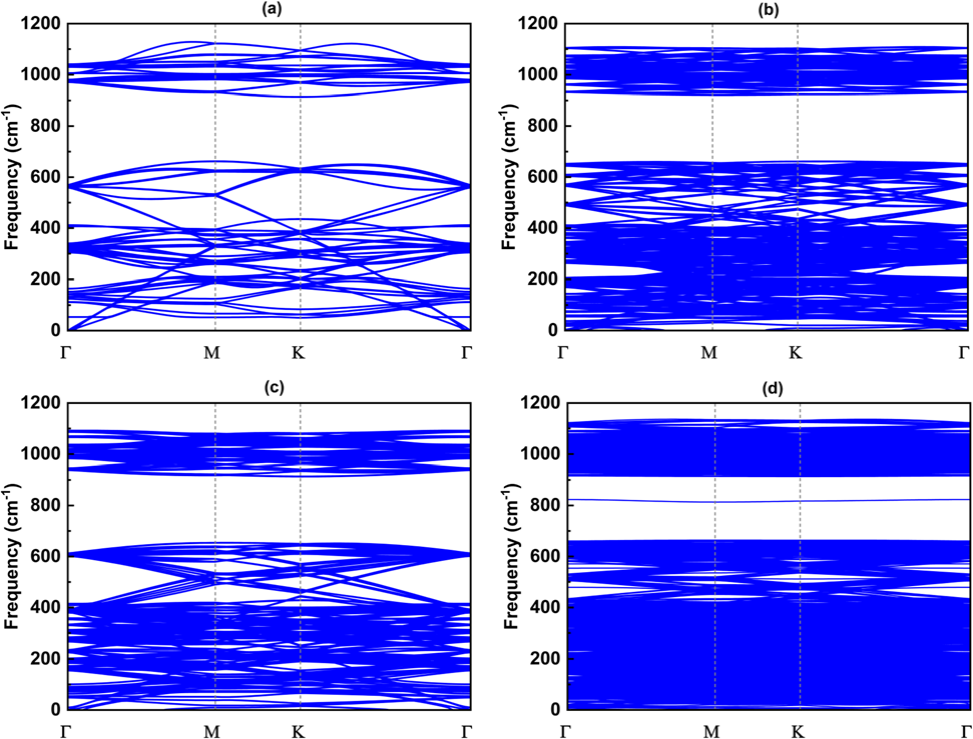
The phonon band with respect to four twist angles: (a) 21.8°, (b) 17.9°, (c) 13.2° and (d) 5.1°.

Since the size of the considered models exceeds our computational capacity, we have used only the (1 × 1) unit cell system for the calculation of the phonon band and the comparison between them. Each twisted unit cell has a different number of atoms and symmetry, which results in a varying number of phonon branches in each configuration. In the case of the 5.1°-twist angle structure, with 508 atoms in a unit cell, the phonon branches in Fig. 8 are so dense that it is difficult to distinguish individual branches. Overall, the phonon dispersion for all the twisted structures, except for the 5.1° twisted angle, shows no imaginary branches, indicating that they are considered thermodynamically stable. In all cases of twist angles, these phonon bands are almost the same. The frequencies of the acoustic bands and lower optical bands are within the range of 0 cm^−1^ to 650 cm^−1^, while the higher optical bands range from 900 cm^−1^ to 1100 cm^−1^. Those four structures all exhibit a phonon band gap ranging from 650 cm^−1^ to 900 cm^−1^, created by the considerable weight difference between two constituent atoms, Si and C. These results are in good agreement with other bare 2D-SiC phonon calculations, with the highest frequency of about 1100 cm^−1^ and a phonon band gap between 650 cm^−1^ and 900 cm^−1^.^31,45^ The unchanged phonon dispersion band indicates that the phononic property of the twisted bilayer SiC model is not affected by the twist angle.

## Conclusions

We have chosen the angles of 21.8°, 17.9°, 13.2° and 5.1° to calculate the influence of the twist angle on the relaxed structure, electronic and phonon band of the twisted bilayer SiC model using DFT. The twist always causes a symmetric distortion of the optimized bilayer structures. Wherever two atoms of the same element form a line orthogonal to the lattice plane, two peaks appear. The interlayer distances of these peaks tend to decrease as the twist angle increases. Meanwhile, the values of the bandgaps are proportional to the twist angles. When the unrelaxed bilayer models are pressed vertically, the band gaps decrease at the same rate regardless of the twist angle. Compared to the pressing effect, the bilayer twist shows less influence on the band gap decrement. However, the effect of the twist angle on the phonon property is trivial and negligible. Since this study can only deal with a limited number of cases, it is necessary to consider more twist angles and extend the model in the future. They would give more special and novel properties of this twisted bilayer SiC system.

## Author contributions

H. T. T. T. conceived the idea of developing the research. H. V. N. conducted the simulation. H. T. T. T. and H. V. N. analysed data, wrote the manuscripts. All authors contributed to the review of the manuscript and approved the final version of the manuscript.

## Conflicts of interest

There are no conflicts to declare.

## Supplementary Material
